# Rational immunogen design to target specific germline B cell receptors

**DOI:** 10.1186/1742-4690-9-S2-O71

**Published:** 2012-09-13

**Authors:** J Jardine, O Kalyuzhniy, T Ota, A McGuire, S Menis, J Julien, E Falkowska, S MacPherson, M Jones, DR Burton, IA Wilson, L Stamatatos, D Nemazee, WR Schief

**Affiliations:** 1 The Scripps Research Institute, San Diego, CA, USA; 2 Seattle BioMed, Seattle, WA, USA

## Background

VRC01 and a number of other broad and potently neutralizing CD4 binding site antibodies have been isolated from HIV positive individuals. These antibodies utilize VH1-2 and make the majority of their contacts via the framework portion of the heavy chain. Recently, it has been noted that the germline precursors to these VRC01-like antibodies do not bind to HIV Env nor does Env stimulate B cell lines expressing these germline precursors. This lack of interaction between germline antibodies and Env may represent a significant block for re-elicitation of these antibodies.

## Methods

We engineered a modified Env to have affinity for the VH1-2 germline antibodies. We believe this antigen will selectively activate B cells that have the potential to elicit VRC01-like antibodies.

Homology modeling and computational protein interface design was used to predict mutations to modify GP120 to have affinity for the VH1-2 germline antibodies. Mutations identified during the computational design were used to generate directed libraries that were screened on the surface of yeast to optimize binding against the germline predicted precursors of several VRC01-like antibodies as well as their mature counterparts.

## Results

Using the strategy outlined above, we have modified a GP120 outer domain to have sub-micromolar affinity for several VH1-2 germline antibodies while maintaining high affinity for the VRC01-like matured antibodies. We have shown in a cell-based assay that, when multimerized, the engineered immunogen stimulates B-cell lines expressing germline VRC01 and other VH1-2 germline antibodies.

## Conclusion

Our immunogen offers a novel approach to re-elicit VRC01-like antibodies. We have demonstrated proof of principle that immunogens can be rationally directed to target specific germline B cell receptors. If this approach proves successful, it could become a generally applicable strategy to selectively activate desirable antibodies when creating new vaccines.

